# Progress and applications of infrared thermography in individuals with intestinal stomas: a scoping review

**DOI:** 10.3389/fonc.2026.1796028

**Published:** 2026-04-20

**Authors:** Wen Xu

**Affiliations:** 1 Department of General Surgery, Nanjing Hospital of Traditional Chinese Medicine, Nanjing, China; 2 School of Nursing, Nanjing University of Chinese Medicine, Nanjing, China

**Keywords:** infrared thermography, intestinal stoma, peristomal skin complications, stoma care, tele-nursing

## Abstract

**Background:**

Intestinal stoma care requires continuous nursing assessment and timely management of peristomal skin complications (PSCs) and perfusion-related risks, yet routine evaluation still relies predominantly on visual inspection and subjective symptom reporting. Infrared thermography (IRT) provides non-contact, visualized temperature monitoring and has been explored across wound and postoperative settings as an objective adjunct for early risk signaling. This review outlines the theoretical foundation of IRT, discusses acquisition and interpretation considerations, and summarizes current evidence in contexts relevant to intestinal stomas, along with implementation-oriented implications for nurse-led stoma care.

**Methods:**

We conducted a structured literature search in Chinese databases, including China National Knowledge Infrastructure (CNKI), Wanfang Database, and China Science and Technology Journal Database (VIP), and in English-language databases (PubMed, CINAHL, Cochrane Library, Web of Science, and Embase) from inception through February 18, 2026. Controlled vocabulary terms and free-text keywords were combined for thermography and stoma/peristomal concepts, supplemented by wound, postoperative monitoring, and perfusion/ischemia-related terms. Two reviewers independently screened titles/abstracts and full texts using predefined eligibility criteria, with adjudication by a third reviewer when needed. Findings were synthesized narratively due to heterogeneity.

**Results:**

Nine studies were included, spanning postoperative infection/delayed healing monitoring, perfusion/ischemia-related evaluation, abdominal thermographic mapping, stimulated thermography for tissue differentiation, and mobile thermography with algorithmic analysis. Across studies, temperature-difference metrics (e.g., temperature difference, ΔT) and pattern-based interpretation were commonly used, while devices, acquisition conditions, and reported thermal metrics varied substantially.

**Conclusion:**

Current evidence supports feasibility of IRT as a non-contact adjunct for temperature-pattern monitoring in contexts relevant to intestinal stoma care, but robust stoma-specific validation remains insufficient. Standardized acquisition procedures, interpretable thresholds, and pathway-level integration into nurse-led decision-making are key prerequisites for translation. Future implementation-ready studies with clinically meaningful endpoints and reproducibility testing are required to define when IRT adds value beyond routine assessment and how it can be embedded safely into real-world stoma care pathways.

## Introduction

Colorectal cancer remains a major global health problem, with an estimated 1.93 million new cases and 0.94 million deaths worldwide in 2020 (1). In China, national statistics likewise show a substantial and rising burden in both incidence and mortality (2). For many patients with colorectal cancer and other intestinal diseases, temporary or permanent intestinal stoma is an essential treatment strategy and often marks the beginning of long-term rehabilitation needs (3). Taken together, intestinal stoma care represents a sustained, system-level challenge spanning acute treatment to long-term follow-up.

Stoma care is clinically important and inherently interdisciplinary. It relies on nurse-led assessment, education, and continuity of care, while also depending on appropriate pouching systems, skin protection materials, and timely complication management across settings. Peristomal skin complications (PSCs) are common and can meaningfully impair comfort, daily function, and quality of life (3). They also create measurable clinical and economic burden for health systems, particularly in the early period after ostomy formation (4). This burden makes prevention and early recognition of peristomal skin problems a core nursing priority.

A key contributor is moisture-associated skin damage around the stoma, often referred to as peristomal moisture-associated skin damage (PMASD), which has distinct mechanisms and management implications in peristomal skin (5). In practice, PMASD is influenced by multiple patient-, device-, and care-related factors, which makes prevention and monitoring challenging and variable across individuals (6). Therefore, stoma care needs assessment approaches that are both standardized and sensitive to early, subtle change.

Despite growing emphasis on standardization, routine peristomal assessment still relies predominantly on visual inspection and subjective symptom reporting. Structured tools such as the Ostomy Skin Tool (OST) improve documentation and grading but remain anchored in visible signs and rater interpretation (7). The updated OST 2.0 strengthens usability and validation, yet it does not fully solve the challenge of detecting early inflammatory or perfusion-related changes before overt erythema, erosion, or ulceration becomes apparent (8). This highlights a persistent gap: early-risk signals may exist before conventional assessment can reliably capture them.

After discharge, continuity becomes even more difficult; remote imaging approaches for peristomal lesion assessment have been explored, but the diagnostic process still depends primarily on what is visually captured and how consistently it is interpreted (9). Meanwhile, digital health solutions for continuous care and self-management are expanding in stoma populations, creating practical opportunities—and expectations—for technology-enabled monitoring in nursing workflows (10). These developments increase the urgency of identifying objective, scalable adjuncts that can operate across care settings.

Against this background, infrared thermography (IRT) is of interest as an objective, non-contact adjunct because skin-surface temperature patterns may reflect underlying inflammation, moisture-related irritation, and perfusion changes that are not yet clearly visible. Evidence from wound care and postoperative monitoring has summarized IRT’s potential roles as well as key protocol issues affecting reliability and usability (11). A scoping review of point-of-care IRT devices across wound types further highlights both feasibility and the persistent need for standard acquisition and interpretation rules (12). This broader evidence base supports biological plausibility and feasibility, while underscoring that standardization is prerequisite for clinical translation.

In surgical contexts, thermographic profiles have been investigated for early recognition of delayed wound healing and surgical site infection (SSI), suggesting potential value for earlier risk stratification than routine clinical recognition in some settings (13, 14). Recent studies also explore mobile thermal imaging combined with machine learning to support SSI detection, reflecting a broader trend toward portable, algorithm-assisted assessment (15, 16). These findings suggest that IRT may contribute when it is embedded into reproducible workflows and decision logic rather than used as images alone.

For intestinal stoma care, the major unresolved gap is not whether IRT can generate images, but whether it can be translated into actionable, nurse-led decision pathways with clear thresholds, defined response steps, and clinically meaningful outcomes. Related evidence from gastrointestinal surgery indicates that thermal patterns can reflect perfusion compromise (e.g., bowel ischemia), supporting biological plausibility for perfusion-related applications (17). Thermographic mapping of the abdomen has also been reported in populations after enterostoma, suggesting that abdominal thermal patterning may be measurable in relevant postoperative contexts (18). However, direct stoma-specific clinical validation and pathway-level evidence remain limited, leaving uncertainty about when and how IRT should change care.

In the era of nurse-led, technology-enabled care, intestinal stoma management lies at the critical intersection of oncology, wound care, and tele-nursing. Effective peristomal skin assessment directly impacts patient comfort, independence, quality of life, and healthcare costs. Despite advances in digital health tools, early detection of subclinical inflammation or perfusion changes remains challenging with conventional visual methods alone.

Accordingly, this review synthesizes current evidence on IRT relevant to intestinal stomas and peristomal assessment, consolidates implementation-ready workflows and key acquisition/interpretation parameters, and defines research priorities for individualized, nurse-led stoma care. Given heterogeneity in devices, protocols, and thermal metrics—and the scarcity of stoma-specific validation—future studies should standardize methods, prospectively test clinically meaningful outcomes, and evaluate real-world pathway integration before routine adoption (4, 12).

## Materials and methods

### Research questions and PCC framework

To align with the objectives of a scoping review, we formulated the following research questions: (1) What IRT acquisition protocols and interpretation approaches have been reported in contexts relevant to intestinal stoma and peristomal skin assessment? (2) What types of clinical contexts and use cases have been studied, and what outcomes or clinical implications have been reported? (3) What implementation-oriented workflow elements can be extracted to inform pathway-level integration of IRT into stoma care?

We used the PCC framework to define the scope (19). Population: individuals with intestinal stomas and/or populations in closely related clinical contexts where findings may plausibly inform stoma care. Concept: infrared thermography/thermal imaging as a non-contact method to generate thermograms or temperature maps for skin/wound assessment, infection/delayed healing monitoring, inflammation-related change detection, or perfusion/viability screening, including temperature-difference (ΔT) and pattern-based interpretation. Context: perioperative and post-discharge care settings relevant to nursing assessment and continuity of care.

### Literature search strategy

This scoping review was conducted and reported in accordance with the PRISMA extension for Scoping Reviews (PRISMA-ScR) (19). We conducted a structured literature search to identify publications on IRT relevant to intestinal stomas, peristomal assessment, and related clinical contexts (e.g., wound assessment, postoperative monitoring, and perfusion/ischemia evaluation). Searches were performed in China National Knowledge Infrastructure (CNKI), Wanfang Database, China Science and Technology Journal Database (VIP) for Chinese-language literature, as well as PubMed, CINAHL, Cochrane Library, Web of Science, and Embase for English-language literature. The search timeframe spanned from inception through February 18, 2026. The search strategy combined controlled vocabulary terms and free-text terms. Key concepts included thermography terms (“infrared thermography,” “thermal imaging,” “thermography,” “infrared imaging”) and stoma/peristomal terms (“intestinal stoma,” “ostomy,” “enterostoma,” “peristomal,” “peristomal skin”), supplemented by complication/assessment and related-context terms (e.g., “peristomal dermatitis,” “skin complications,” “wound,” “surgical site infection,” “ischemia,” “perfusion,” “viability,” “monitoring,” “telemedicine,” “remote imaging”). Reference lists of key reviews and included studies were also screened manually to identify additional eligible records.

Search query using PubMed as an example: (“Thermography”[Mesh] OR “infrared thermography”[Title/Abstract] OR “infra-red thermography”[Title/Abstract] OR thermograph*[Title/Abstract] OR “thermal imaging”[Title/Abstract] OR “infrared imaging”[Title/Abstract]) AND (“Ostomy”[Mesh] OR ostom*[Title/Abstract] OR stoma*[Title/Abstract] OR enterostom*[Title/Abstract] OR colostom*[Title/Abstract] OR ileostom*[Title/Abstract] OR urostom*[Title/Abstract] OR peristomal[Title/Abstract] OR “peristomal skin”[Title/Abstract] OR peristoma[Title/Abstract]).

### Eligibility criteria

Studies were eligible if they (1) used IRT/thermal imaging to generate thermograms or temperature maps, and (2) reported clinically interpretable methods and/or findings relevant to skin/wound assessment in contexts applicable to intestinal stoma care (including peristomal/periwound assessment, postoperative incision monitoring such as SSI/delayed healing, or perfusion/ischemia-related evaluation). We included empirical studies of any design published in English or Chinese, with full text available.

Studies were excluded if they (1) did not use IRT/thermal imaging, (2) addressed applications unrelated to wound/skin care with no care-relevant interpretation, (3) were engineering-only/algorithm-only reports without clinically usable acquisition details or interpretable clinical outputs, or (4) were non-empirical publication types (editorials, commentaries, letters, narrative opinions, and non-systematic overviews). Conference proceedings/abstracts were excluded. For duplicate or overlapping reports, the most complete and most recent report was retained.

### Study selection and data extraction

All records retrieved from database searches were imported into NoteExpress for reference management. Duplicate records were identified and removed using the software’s automated function, followed by manual checking when necessary. Screening was conducted in two stages. First, two reviewers (ZX and HS) independently screened titles and abstracts against the eligibility criteria. Second, the same reviewers independently assessed the full texts of potentially eligible articles for final inclusion. Disagreements at either stage were resolved through discussion; if consensus could not be reached, a third reviewer (XW) adjudicated.

Following study inclusion, data were extracted and recorded in a structured form developed *a priori*. When key information was unclear or incompletely reported, we extracted available details as stated by the authors and noted the missing elements. The final dataset was checked for consistency across reviewers before synthesis.

### Critical appraisal

Consistent with scoping review methodology and the aim to map the breadth, characteristics, and implementation implications of the evidence rather than to estimate effect sizes, we did not conduct a formal critical appraisal/risk-of-bias assessment of included studies. Instead, we recorded key methodological features that influence interpretability and reproducibility and incorporated these considerations into the interpretation of evidence gaps.

### Synthesis approach

Given heterogeneity in study designs, devices, acquisition conditions, and reported thermal metrics, findings were synthesized using a descriptive-numerical summary and narrative thematic synthesis. First, we summarized the distribution of study contexts, device types, and application categories. Second, we organized extracted evidence into implementation-oriented themes relevant to stoma care, including: (i) postoperative monitoring for infection risk/delayed healing, (ii) perfusion/ischemia-related evaluation, (iii) thermographic mapping and stimulated thermography, and (iv) mobile thermography with algorithmic analysis. Across themes, we emphasized reproducible acquisition conditions, interpretable thermal metrics and workflow elements that could support nurse-led decision pathways, while explicitly noting where evidence was stoma-specific versus adjacent-context and therefore hypothesis-generating for stoma care.

## Results

**Figure 1:**
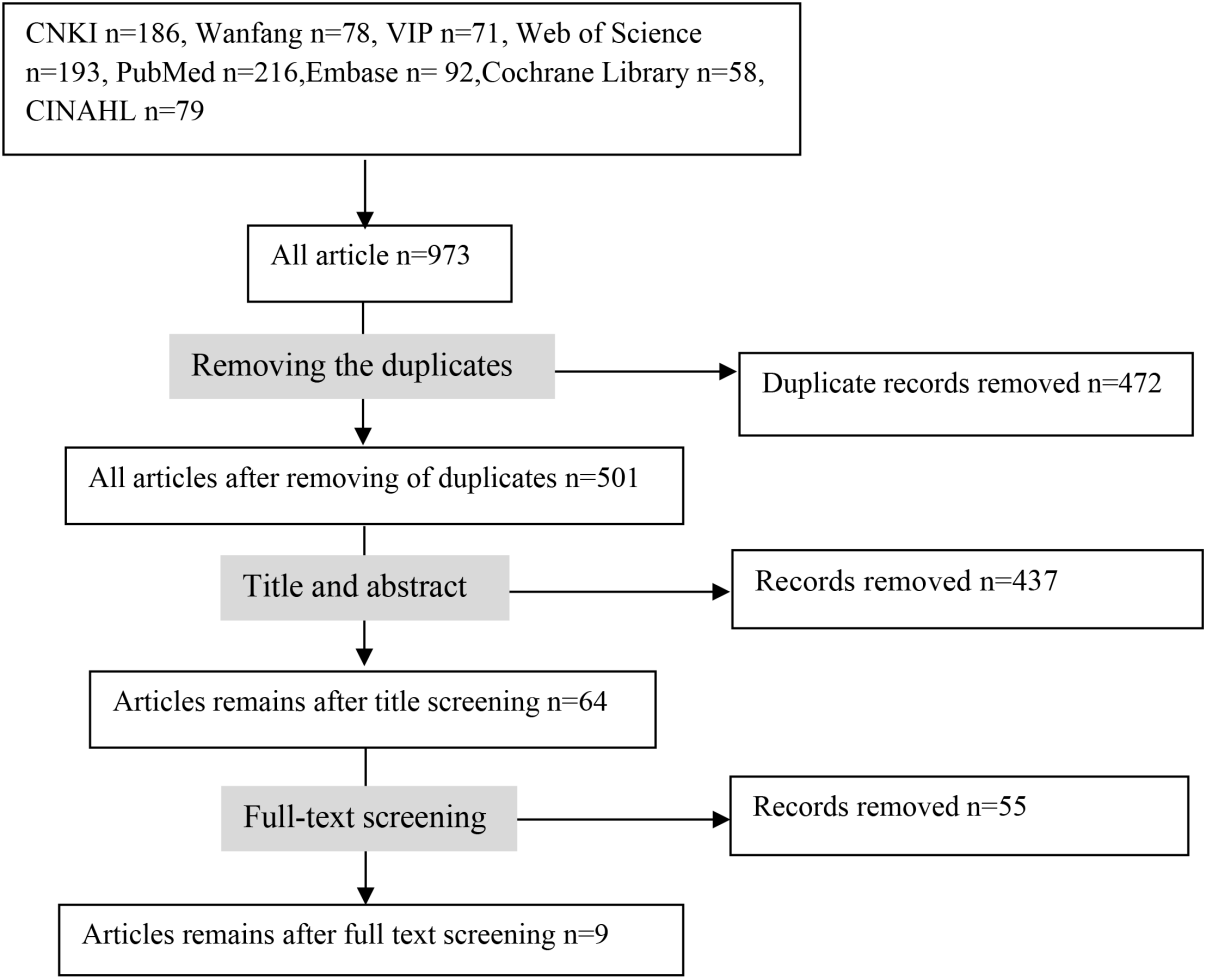
PRISMA-ScR flow diagram of the study selection process.

**Figure 2:**
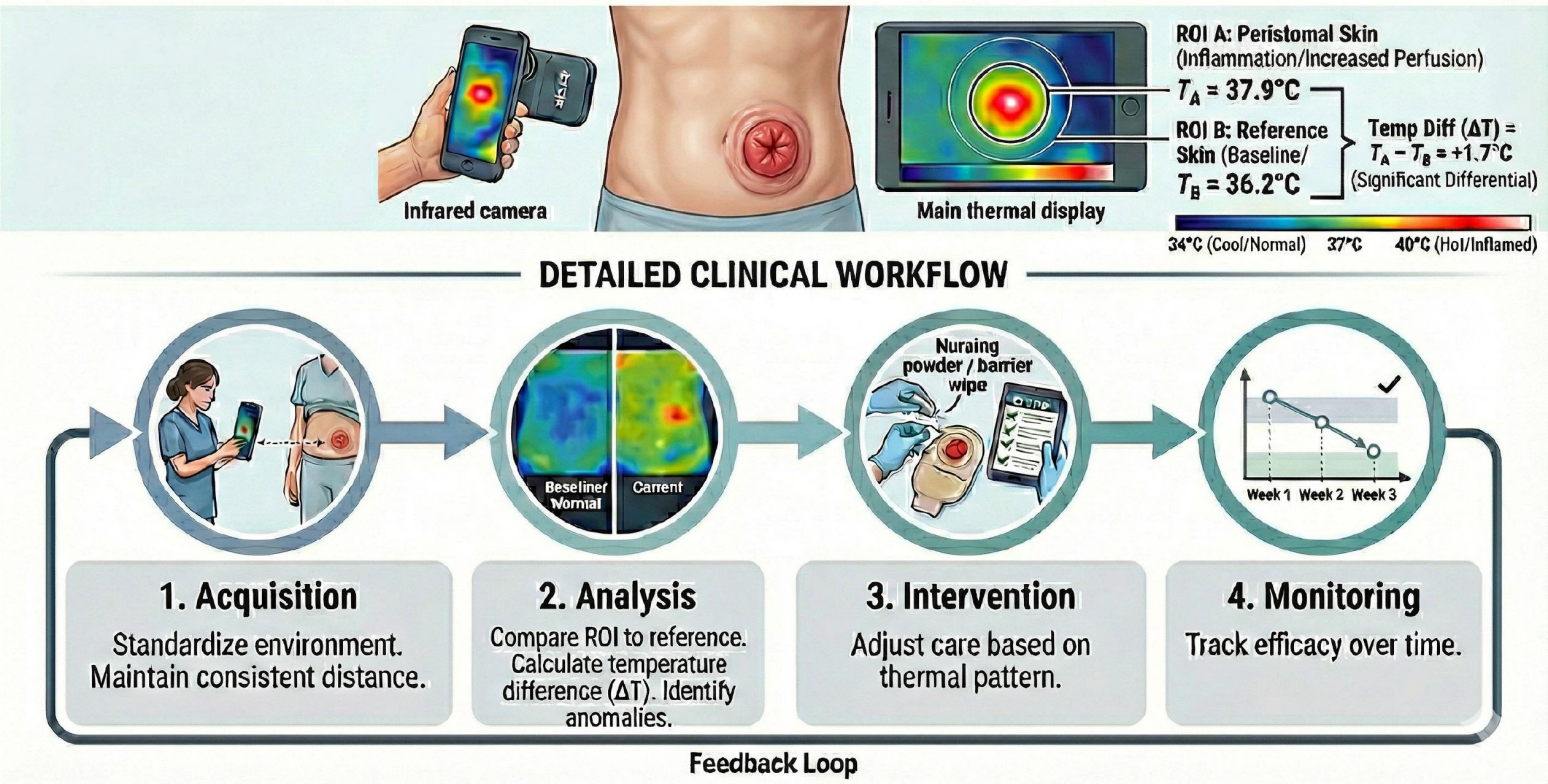
Schematic diagram of infrared thermography (IRT) principle and clinical workflow in stoma care.

After screening, nine studies were included in the synthesis (**Figure 1**). A practical, implementation-oriented overview is presented in **Figure 2**. The key characteristics of the included studies are summarized in **Table 1**.

**Table 1:** General characteristics of the included studies(n=9).

First author (year)	Country	Sample size	Target population/Organ of interest	Appliance
Childs (13) (2019)	UK	53	Obese women (BMI ≥30) post-caesarean	Clinical IR thermography
Siah (14) (2019)	Singapore	60	Post-enterostoma closure surgical wounds	Standard clinical IR camera
Prey (16) (2024)	USA	177	General surgery incisions	FLIR ONE PRO LT (mobile phone)
Brooks (17) (2000)	USA	4 pigs	Porcine mid-jejunum (bowel ischemia)	Thermal imaging camera (tripod)
Siah (18) (2015)	Singapore	30 healthy +10 pts	Healthy abdomen + post-enterostoma wounds	FLIR T640
Li (20) (2023)	China	40	Thoracic surgical incisions	Smartphone InfiRay T3Pro
Childs (21) (2016)	UK	20	Post-caesarean abdomen + infected wounds (scar line)	FLIR T450sc
Fujita (22) (2013)	Japan	1 (case)	Post-Nuss pectus bar site (chest wall)	FLIR B60 handheld
Riquet (23) (2016)	France	12	Surgical/wound scars (scar vs peri-scar tissue)	FLIR A20

First author (year)	Methodology	Key data	Clinical implications	Changes in management
Childs (13) (2019)	Prospective test-accuracy (day 2 + home days 7/15/30)	Wound-abdomen ΔT (OR 2.25–2.5); 70–79% correct classification	Superior to visual assessment; predicts SSI in high-risk group	Community/home feasible; improves antibiotic decision-making
Siah (14) (2019)	Prospective observational (days 1–4)	Infected wounds significantly cooler; partial warming pattern	Detects delayed healing within 4 days before discharge	Enables early intervention; supports nurse-led wound assessment
Prey (16) (2024)	U-net ML model for wound segmentation (RGB+thermal)	Pixel accuracy 89–92%, Jaccard 64–66%	Excellent wound boundary detection; potential for early SSI	Low-cost mobile tool; ideal for community /telemedicine
Brooks (17) (2000)	Prospective animal model; 5 min + 2 h warm ischemia	100% sensitivity for necrosis; black/white demarcation	Safe non-invasive adjunct for bowel viability assessment	Repeatable during surgery; useful when visual/Doppler uncertain
Siah (18) (2015)	Thermographic mapping (9 quadrants + stoma site)	BMI >25 → lower abdominal temp; infected wounds show cold spots	Baseline reference for abdominal thermography; cold spots flag infection	Distance up to 100 cm reliable; BMI must be considered
Li (20) (2023)	Prospective cohort (daily for 7 days + 1–2 mo follow-up)	Day-4 min temp & ΔT best predictors (sens 91.67%, spec 85.71%)	Non-linear healing trajectory; early prediction of 1–2 mo outcome	Nurse-friendly, portable, visual early-warning system
Childs (21) (2016)	Prospective feasibility; HCS pixel clustering + ΔT profiling	ΔT >2 °C, cold spots along scar, HCS clusters	Cold spots at day 2 predict later SSI; infected wounds cooler	Bedside feasible; dressings removed 30 min prior; highly acceptable to mothers
Fujita (22) (2013)	Case report screening at week 5	Early hot-spot detection before clinical signs	Detects subclinical cellulitis; prevents progression	Rapid, non-invasive screening for subcutaneous implants
Riquet (23) (2016)	Stimulated IRT (ice bag 2 min + 10 min rewarming)	Scars significantly colder; ΔT >0.5 °C visible	Reliable differentiation of scar from healthy tissue	Simple cold-stimulation protocol; useful for scar assessment

### Postoperative monitoring: infection risk and delayed healing

Across postoperative contexts, included studies reported interpretable thermographic ΔT features and pattern changes during early recovery, with measurable classification or predictive performance in selected cohorts. Childs et al. (13) reported wound–abdomen ΔT features associated with SSI risk and 70–79% correct classification, exceeding visual assessment performance in that population. Siah et al. (14) reported cooler infected wounds with partial warming patterns during postoperative days 1–4, supporting early identification of delayed healing before discharge. Li et al. (20) reported day-4 minimum temperature and ΔT as the strongest predictors of later incision-healing outcomes (sensitivity 91.67%, specificity 85.71%) using smartphone-based IRT over 7 days with 1–2 month follow-up. Childs et al. (21) characterized abdominal thermal territories after caesarean section using clustering/ΔT profiling and reported early scar-line cold-spot patterns linked to subsequent infection-related outcomes. Fujita et al. (22) described an early hot-spot pattern at a pectus bar site preceding overt clinical signs.

### Perfusion/ischemia-related evaluation

Across perfusion-oriented evidence, thermal imaging was used to delineate temperature patterns consistent with ischemic risk under controlled conditions, providing objective cues alongside conventional observation. Brooks et al. (17) reported clear thermal demarcation and 100% sensitivity for necrosis under warm ischemia protocols. These findings provide model-based evidence that temperature mapping can reflect bowel viability changes when perfusion is experimentally altered. However, study designs and thresholds were not directly comparable across settings, highlighting the need for standardized acquisition conditions and clinically validated cutoffs in human stoma-related applications.

### Thermographic mapping and stimulated thermography

Studies using mapping and stimulated protocols reported structured abdominal thermal territory patterns and tissue-response differences under defined acquisition approaches, informing interpretation beyond single-point temperature readings. Siah et al. (18) reported quadrant-based abdominal mapping including the stoma site, lower abdominal temperature in BMI >25, cold-spot patterns in infected wounds, and feasibility up to 100 cm acquisition distance. Riquet et al. (23) reported stimulated IRT differentiation of scar versus peri-scar tissue after cold stimulation, with scars colder and ΔT >0.5 °C visually detectable during rewarming. Together, these studies illustrate how baseline territory mapping and dynamic provocation can support pattern-based interpretation under standardized protocols.

### Mobile thermography with algorithmic analysis

Algorithm-assisted approaches applied mobile thermal imaging to support scalable image-based assessment, reporting quantitative performance metrics rather than purely qualitative interpretation. Prey et al. (16) used RGB+thermal imaging for wound segmentation and reported pixel accuracy 89–92% and Jaccard 64–66%. This study emphasizes feasibility of integrating thermography with automated image processing in mobile settings, which may be relevant to community follow-up workflows. As with other domains, generalizability depends on external validation, event rates, and standardized acquisition to ensure that model performance is reproducible across devices and environments.

## Discussion

### Principal findings and relevance to stoma care

Included studies reported that IRT can capture clinically interpretable ΔT features and pattern changes in postoperative and wound-related contexts, with quantifiable performance signals in selected cohorts (e.g., test-accuracy or predictive metrics) (13, 14). Smartphone-based implementation feasibility and longitudinal monitoring were also reported, supporting the practical capture of serial thermograms in routine workflows (20). Mapping and stimulated protocols further demonstrated that abdominal thermal territories and tissue-response patterns can be characterized under defined acquisition approaches (18, 23). Therefore, the central contribution of this evidence base is to frame IRT as an adjunctive monitoring signal that must be operationalized into decisions rather than treated as a stand-alone diagnostic output.

### Clinical translation: from thermograms to nurse-led decisions

Evidence supports positioning IRT within nurse-led action pathways that combine structured assessment and thermographic triggers. Structured tools such as OST and OST 2.0 provide standardized grading of peristomal skin lesions, but remain anchored in visible signs and rater interpretation; adding thermographic documentation can strengthen continuity across shifts and follow-up records (7, 8). Mobile applications have been explored to support prevention and treatment of peristomal skin complications, providing a practical route for technology-enabled education and follow-up support (24). Telemedicine-based assessment of wound complications provides a precedent for remote monitoring models, but decision quality depends on standardized inputs and escalation logic rather than images alone (25). Therefore, IRT implementation should be designed as a closed-loop workflow—trigger, verify acquisition conditions, intervene (e.g., sealing optimization/barrier protection vs escalation), and reassess—so that thermal findings translate into reproducible nursing actions.

### Interpretation and standardization: ΔT, trends, and artifacts

Evidence supports using relative ΔT and temporal trends as the primary interpretive basis because thermograms reflect perfusion and inflammatory metabolism while remaining sensitive to acquisition conditions and environment (11, 12). Feasibility protocols reported in postoperative thermography include practical steps such as standardized timing and adequate exposure/acclimation, which can inform stoma-adapted operating procedures (21). In stoma settings, moisture, folds, and adhesive residues increase the risk of artifacts, and baseline pattern modifiers (e.g., BMI) need to be considered when interpreting abdominal territories (18). Therefore, stoma-specific SOPs should specify acquisition timing, distance/angle, reference-area selection, minimum documentation items, and artifact checks before thermographic findings are used for escalation.

### Perfusion-related assessment: adjunctive positioning and validation needs

Evidence supports biological plausibility for perfusion-related use of thermal imaging, with controlled-model findings showing clear demarcation patterns under ischemia protocols (17). In clinical practice, bowel perfusion assessment may rely on established approaches such as near-infrared fluorescence; large-scale trial evidence in colorectal surgery highlights this as a mature comparator for perfusion-directed decision-making (26). Reviews of intestinal viability techniques emphasize that different modalities carry distinct trade-offs and that clinical validation depends on reference standards and outcome definitions (27). Therefore, IRT should be positioned as an adjunctive screening signal that triggers reassessment and multidisciplinary escalation, and future stoma-specific studies should validate ΔT/trend rules against clinical observation and established perfusion assessment methods.

### Continuity of care, self-management, and digital platforms

Implementation requires aligning IRT with continuity-of-care models because post-discharge monitoring gaps and variable self-management contribute to recurrent skin problems. A scoping review of mobile health in continuous stoma care summarizes the range of mHealth-enabled follow-up strategies and provides context for integrating thermograms into existing digital workflows (10). Evidence also links PMASD with independence in pouching system changes, supporting triage models that incorporate self-care capability alongside objective signals (28). Integrative evidence has summarized nursing-led strategies to prevent and manage peristomal skin complications, emphasizing standardized assessment, barrier protection, and patient education as core components of stoma care (29). Remote imaging approaches for peristomal lesion assessment demonstrate feasibility but also underscore that image-based monitoring still relies on standardized capture and consistent interpretation (9). Therefore, a tiered nurse-led follow-up pathway should combine visible-light images, thermograms, leakage history, and structured scoring while explicitly incorporating self-management capacity into risk stratification and follow-up intensity.

### Data governance and implementation safety

Implementation requires explicit data governance because peristomal images and thermograms constitute sensitive health information. A best-evidence summary on self-management for peristomal skin complications supports the need for standardized education and documentation elements, which can be extended to include thermogram capture and interpretation cues (30). Regulatory and compliance sources emphasize that mobile platforms handling sensitive health information require controlled access, secure transmission, and auditable workflows (31, 32). Therefore, deployment in tele-follow-up should adopt end-to-end security measures (encryption, access control, retention policies) and consider centralized specialist interpretation models to reduce privacy risk while improving interpretive consistency.

### Evidence gaps and research priorities

Evidence indicates that stoma-specific IRT validation remains limited and that the current literature is heterogeneous in devices, acquisition conditions, and thermal metrics, constraining cross-setting comparability (11). Clinical burden data show that PSCs carry measurable economic impact, strengthening the case for evaluating whether IRT-enabled pathways reduce utilization and costs rather than only improving image-based detection (4). Risk prediction work for PMASD in stoma populations suggests an opportunity to integrate objective thermographic signals into risk models, but prospective validation is required to avoid overfitting and false escalation (5). Therefore, future studies should adopt implementation-ready designs with standardized acquisition, predefined action thresholds, clinically meaningful endpoints (PSC/PMASD incidence/severity, leakage outcomes, pain, utilization, costs), and reliability testing under real-world conditions.

### Study limitations

This review has several limitations. First, the available evidence directly focused on intestinal stomas and peristomal assessment is limited; consequently, part of the synthesis relies on evidence from adjacent clinical contexts (e.g., postoperative incision monitoring, wound assessment, and perfusion/ischemia-related evaluation). Second, heterogeneity across devices, acquisition conditions, and reported thermal metrics limited comparability and precluded quantitative pooling. Third, although we applied predefined eligibility criteria and a structured screening process, study selection and narrative synthesis may still introduce selection bias and interpretation bias, as is inherent to non-meta-analytic reviews. Fourth, publication bias cannot be excluded, and studies with negative or inconclusive findings may be underrepresented. Finally, because reporting of acquisition parameters and reference standards was inconsistent across studies, the strength of practice-oriented conclusions remains constrained. Therefore, conclusions should be interpreted as hypothesis-generating and implementation-oriented rather than definitive clinical guidance.

## Conclusion

Infrared thermography (IRT) provides a non-contact, objective adjunct for visualized temperature monitoring that meaningfully complements conventional visual inspection in intestinal stoma care. Across related clinical contexts, IRT demonstrates feasibility for early postoperative monitoring, perfusion screening, and tele-nursing support. Nevertheless, evidence is heterogeneous and largely indirect, with insufficient stoma-specific validation; standardized acquisition, actionable thresholds, and nurse-led pathway integration remain to be established. The ultimate goal is early detection of subclinical thermal changes—cold-spot/hot-spot patterns (ΔT >1.5–2.0 °C) or persistent hypothermic areas—acting as a practical “thermal early-warning” layer to trigger immediate clinical actions, particularly in high-risk groups (patients with BMI >25, early postoperative phase, or limited self-care capacity), thereby preventing progression to peristomal skin complications (PSCs) and peristomal moisture-associated skin damage (PMASD) while reducing pain, readmissions, and costs.

To translate this potential into practice, future implementation-ready studies should focus on the following clinically meaningful endpoints: (i) incidence and severity of PSCs/PMASD using validated tools (e.g., OST/OST 2.0 with longitudinal change); (ii) time-to-detection and time-to-intervention compared with standard care; (iii) leakage-related outcomes, patient-reported pain, and self-management performance; (iv) healthcare utilization and cost-effectiveness; (v) reliability and reproducibility of acquisition and interpretation under standardized protocols.
